# CPAP improves sleep stability and attenuates acute nocturnal hypertension (NBPF) in OSA, with maximal benefits in severe cases

**DOI:** 10.3389/fneur.2025.1587127

**Published:** 2025-07-07

**Authors:** Liangcai Yu, Lan Shu, Simin Gao, Lin Li

**Affiliations:** ^1^West China School of Public Health and West China Fourth Hospital, Sichuan University, Chengdu, China; ^2^Zigong Fourth People's Hospital, Zigong, China

**Keywords:** continuous positive airway pressure, sleep structure, obstructive sleep apnea, nocturnal blood pressure, acute blood pressure surge events

## Abstract

**Objective:**

To investigate the effects of continuous positive airway pressure (CPAP) therapy on sleep architecture, particularly the microarousal index (MAI), and the frequency of nocturnal acute blood pressure surge events (NBPF) in patients with obstructive sleep apnea (OSA), and to analyze the association between improvements in sleep architecture (especially reduced MAI) and decreased NBPF.

**Methods:**

A retrospective analysis was conducted on 477 patients diagnosed with OSA at the Sleep Medicine Center of West China Fourth Hospital, Sichuan University, between January 2021 and January 2023, who received CPAP therapy (mean age 42.70 ± 10.90 years; mild: 92, moderate: 108, severe: 277). Comparisons were made of polysomnography (PSG)-monitored sleep architecture parameters (N1%, N2%, N3%, R%, MAI) and nocturnal blood pressure indices (SBP, DBP, NBPF) before and after CPAP treatment. NBPF was defined as the number of events per hour where nocturnal systolic blood pressure (SBP) increased by >12 mmHg.

**Results:**

(1) Baseline characteristics: N1%, N2%, SBP, DBP, and NBPF significantly increased, while R% and N3% significantly decreased with increasing severity of OSA (*p* < 0.05). (2) Overall efficacy: After CPAP treatment, N1% significantly decreased, N3% significantly increased, and DBP and NBPF significantly decreased (*p* < 0.05). (3) Subgroup analysis: All patients experienced significant reductions in MAI, N1%, N2%, and NBPF, and significant increases in R% after CPAP treatment (*p* < 0.05); N3% significantly increased in moderate and severe patients (*p* < 0.05); SBP and DBP improvements were most significant in severe patients (*p* < 0.05). (4) Correlation and linear regression analysis: NBPF was significantly correlated with sleep structure parameters, showing an independent positive correlation with MAI (*β* = 0.375, *p* < 0.001) and an independent negative correlation with N3% (*β* = −0.143, *p* = 0.001).

**Conclusion:**

The first night of positive airway pressure (PAP) therapy significantly improves sleep architecture and effectively reduces nocturnal blood pressure while suppressing acute systolic blood pressure (NBPF) elevations in OSA patients, especially those with severe disease.

## 1 Introduction

Obstructive sleep apnea (OSA) is a systemic disorder characterized by partial or intermittent complete upper airway obstruction during sleep, resulting in hypoxemia and sleep fragmentation that severely disrupts normal ventilation and sleep patterns (1). Patients with OSA commonly exhibit symptoms including daytime hypersomnolence, fatigue, irritability, and difficulty concentrating, as well as nocturnal snoring, breathing pauses, profuse sweating, salivation, gastroesophageal reflux, and nocturia. Epidemiological studies indicate that approximately 936 million individuals aged 30 to 69 years worldwide suffer from OSA, with China accounting for the largest proportion at an estimated 176 million patients (2). A concerning finding is that only 4% of men and 2% of women met diagnostic criteria for OSA (requiring both AHI ≥ 5 events/h and daytime hypersomnolence). However, two-thirds of subjects without sleep apnea (AHI < 5) also reported excessive daytime sleepiness. This diagnostic ambiguity contributes to substantial underdiagnosis and undertreatment of OSA, exposing patients to significantly higher risks of hypertension, cardiovascular disease, heart failure, obesity, metabolic disorders, diabetes, depression, accidents, and stroke (3).

The integrity of sleep architecture is crucial for cardiovascular homeostasis. Polysomnography (PSG) has confirmed that patients with OSA exhibit characteristic sleep architecture disturbances, specifically a significant reduction in the proportion of deep sleep (N3 stage) and rapid eye movement (REM) sleep, an increase in the proportion of light sleep (N1/N2 stages), and a marked elevation in the microarousal index (MAI). This sleep fragmentation exerts multifaceted adverse effects on the cardiovascular system by activating the sympathetic nervous system and inducing oxidative stress and systemic inflammatory responses, with particularly pronounced disruption of blood pressure regulation (4–6).

OSA is recognized as an independent risk factor for hypertension, particularly refractory hypertension (7). Beyond elevating overall blood pressure levels, growing evidence indicates that OSA poses a serious threat to the dynamic stability of blood pressure during sleep. Notably, nocturnal acute systolic blood pressure surge events serve as sensitive indicators of sympathetic nervous system overactivation and cardiovascular stress (8–10). The nocturnal blood pressure fluctuation index (NBPF) examined in this study specifically refers to the number of events per hour during nocturnal sleep where systolic blood pressure increases by >12 mmHg. NBPF constitutes a key metric for assessing blood pressure instability during sleep. These frequent acute systolic blood pressure surges are directly linked to respiratory event-related microarousals/terminations and hypoxemia-reoxygenation cycles, reflecting paroxysmal surges in sympathetic nervous activity and cardiovascular stress during sleep.

Continuous positive airway pressure (CPAP), as the first-line therapy for moderate-to-severe OSA, effectively maintains upper airway patency, eliminates apnea/hypopnea events, reduces intermittent hypoxemia, and suppresses event-related microarousals (11). Studies have confirmed that CPAP reduces 24-h average blood pressure and office blood pressure in OSA patients (12, 13). Recent studies demonstrate that CPAP efficacy is closely associated with airway stability. Saha et al. (14) confirmed through computational fluid dynamics modeling that a pressure of 9 cmH_₂_O (882.6 Pa) optimizes airflow distribution in the nasopharynx and mitigates collapse risk. Huang et al. (15) further revealed that BiPAP (bilevel positive airway pressure) settings require individualization to prevent distal airway overdistension. However, research on CPAP’s efficacy in improving the most characteristic nocturnal acute blood pressure abnormality in OSA patients—namely, frequent acute systolic blood pressure surge events—remains limited and requires further investigation.

Therefore, this study aimed to accurately investigate, through a large-sample retrospective analysis, the impact of CPAP therapy on OSA patients regarding the following core objectives: (1) To assess the short-term improvement effects of CPAP therapy on sleep architecture parameters (with a particular focus on the microarousal index, MAI) and the key nocturnal blood pressure event metric—namely, the number of events per hour with systolic blood pressure increase >12 mmHg (NBPF)—in the overall OSA cohort and across subgroups of varying severity (mild, moderate, severe). (2) To specifically analyze the correlation between changes in the microarousal index (MAI) and changes in the frequency of acute systolic blood pressure surge events (NBPF), and to rigorously examine whether MAI serves as an independent predictor of NBPF. (3) To definitively reveal the differential efficacy patterns of CPAP in reducing microarousals (decreased MAI) and lowering acute blood pressure surge events (decreased NBPF) across OSA severity subgroups, with particular focus on evaluating its superior efficacy in patients with severe OSA.

The anticipated contributions of this study are threefold: (1) To provide the first large-scale systematic evidence of CPAP-induced suppression of characteristic nocturnal acute systolic blood pressure surge events (NBPF) in OSA patients. (2) To empirically establish, for the first time in clinical research, a robust association and independent predictive effect between reduced microarousals (decreased MAI) and decreased acute blood pressure events (reduced NBPF). (3) Through severity-stratified analysis, to definitively demonstrate the superior efficacy of CPAP in stabilizing nocturnal blood pressure specifically in severe OSA patients, thereby providing crucial evidence for individualized treatment strategies and prognostic assessment.

## 2 Materials and methods

### 2.1 Research design and patients

This study is a cross-sectional observational study. The clinical data of patients with snoring and sleep apnea from June 2022 to June 2024 in the Department of Otorhinolaryngology/Sleep Medicine Center of West China Fourth Hospital of Sichuan University were retrospectively analyzed. All patients were diagnosed with OSA by polysomnography (PSG) and treated with CPAP on the second night, a total of 477 cases.

Inclusion criteria: (1) PSG examination met the above guidelines’ diagnostic criteria, that is, AHI ≥ 5 times/h; (2) 18–65 years old; (3) patients who underwent CPAP treatment and completed pressure titration in our department. Exclusion criteria: (1) patients with basic lung diseases, malignant tumors, severe cardiovascular diseases, mental illness, and pregnancy; (2) patients who could not tolerate CPAP treatment or repeated poor cooperation; (3) PSG monitoring results showed that patients with total sleep time less than 4 h at night.

### 2.2 Methods

#### 2.2.1 Polysomnography

On the first night, all individuals received a PSG evaluation with the SOMNOscreen™ plus PSG + model polysomnography monitor. The detection criteria comprised oral and nasal airflow, blood oxygen saturation, electroencephalogram, electrooculogram, mandibular electromyography, thoracoabdominal respiratory movement, posture, anterior tibial muscle electromyography, blood pressure, and so on. Ask the patient not to nap on the day of monitoring, not to drink coffee, tea, or other liquids that interfere with sleep, and to keep the face, fingers, and other areas clean to ensure smooth development. After all patients arrive at the hospital, they are linked by a professional sleep technician for a full night of sleep monitoring, with a sleep period of at least 7 h. According to the recognized standards of the American Academy of Sleep Medicine (AASM) (16), the identification and scoring were performed. The PSG was interpreted by sleep medicine specialists.

#### 2.2.2 Continuous positive airway pressure

Patients with OSA were followed and treated with CPAP during the second night. All patients received treatment with an automated non-invasive breathing ventilator, APAP mode, a nasal mask, and autonomous pressure titration. The titration pressure for positive airway pressure breathing was adjusted to 4–16 cm H2O (1 cm H2O = 0.098 kPa), and the titration therapy lasted at least 7 h.

### 2.3 Observed indexes

The height and weight of the patients were measured, and the body mass index (BMI) was calculated. Sleep parameters and blood pressure were observed, such as apnea-hypopnea index (AHI), microarousal index (MAI), total sleep time (TST), the percentage of sleep time in each period in total sleep time (R%, N1%, N2% and N3%), systolic blood pressure (SBP), diastolic blood pressure (DBP), and nocturnal blood pressure fluctuation index (NBPF). NBPF was defined as the number of events per hour where nocturnal systolic blood pressure (SBP) increased by >12 mmHg.

### 2.4 Statistical analyses

Excel was used to enter data, while SPSS 25.0 and Stata 14.0 statistical tools were used to process it. The W test for normality was performed, and the information conformed to normal distribution and was expressed as mean ± standard deviation; comparisons between multiple samples were performed by analysis of variance (ANOVA), and comparisons between two groups were performed by the LSD method for multiple comparisons; comparisons before and after treatment were performed by the paired t-test; and the correlation between nocturnal blood pressure and sleep structure was analyzed by Spearman’s analysis, and indicators with significant correlation were included in the multivariate linear regression analyses. All tests were two-sided, and *p* < 0.05 indicated statistical significance.

### 2.5 Technology roadmap

**Figure 1:**
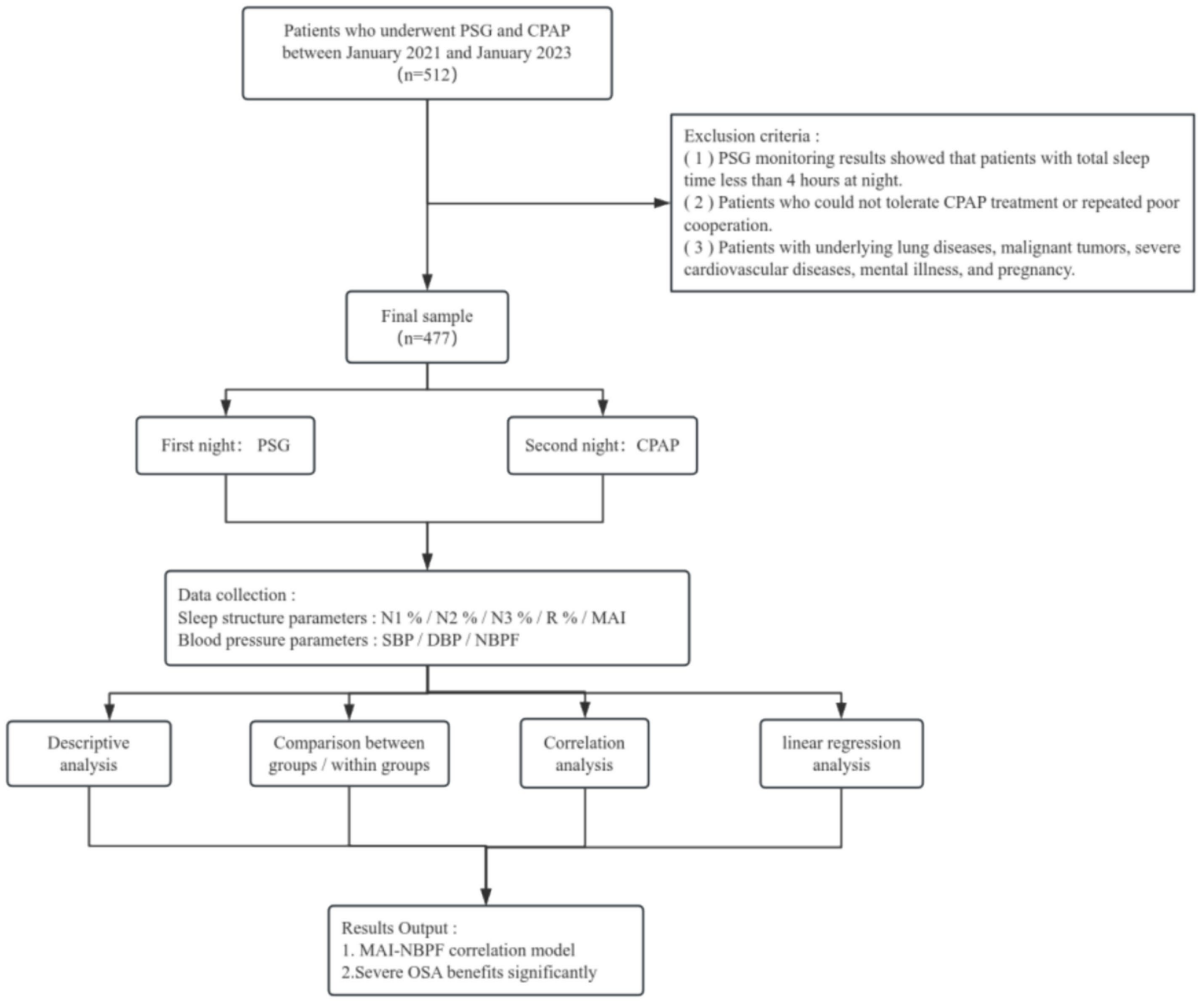
Technical roadmap.

The technical roadmap of this study (Figure 1) is as follows.

## 3 Results

### 3.1 Comparison of demographic characteristics and PSG characteristics of patients with different degrees of OSA

Among the 477 patients with OSA, there were 92 cases of mild OSA, accounting for 19.3%; 108 cases of moderate OSA, accounting for 22.6%; and 277 cases of severe OSA, accounting for 58.1%. The average age of patients was 42.70 ± 10.90 years. As the severity of the disease increased, R% and N3% decreased, while MAI, N2%, N3%, SBP, DBP, and NBPF increased, with the most significant changes observed in severe OSA (Table 1).

**Table 1:** Comparison of demographic and PSG characteristics of OSA patients with different degrees of OSA.

Characteristic	OSA			*F*	*p*
	Mild (*n* = 92)	Moderate (*n* = 108)	Severe (*n* = 277)		
Age/yr.	38.29 ± 11.58	44.58 ± 11.75^a^	43.43 ± 9.94^a^	10.133	<0.001
BMI/(kg/m^2^)	24.52 ± 3.10	25.37 ± 2.34^a^	27.60 ± 3.14^ab^	47.403	<0.001
AHI/h^−1^	9.18 ± 3.03	21.56 ± 4.38^a^	59.81 ± 16.90^ab^	673.124	<0.001
MAI/h^−1^	24.18 ± 10.69	26.63 ± 12.74	44.65 ± 18.24^ab^	84.992	<0.001
TST/m	454.90 ± 71.98	452.68 ± 72.10	459.72 ± 77.03	0.396	0.673
R/%	18.63 ± 5.17	17.22 ± 6.07	15.87 ± 5.42^ab^	9.219	<0.001
N1/%	7.82 ± 3.90	9.41 ± 6.44	13.84 ± 12.64^ab^	15.273	<0.001
N2/%	53.05 ± 10.66	55.06 ± 9.79	59.23 ± 12.34^ab^	12.146	<0.001
N3/%	20.50 ± 10.01	18.31 ± 10.21	11.07 ± 9.81^ab^	41.247	<0.001
SBP/mmHg	109.41 ± 15.77	114.95 ± 12.27^a^	120.75 ± 17.13^ab^	19.027	<0.001
DBP/mmHg	72.74 ± 12.47	76.50 ± 11.18^a^	80.17 ± 12.32^ab^	13.944	<0.001
NBPF/h^−1^	9.95 ± 11.78	13.69 ± 13.51	41.01 ± 30.71^ab^	79.958	<0.001

BMI, body mass index; AHI, apnea-hypopnea index; MAI, micro awakening index; TST, total sleep time; R, rapid eye movement sleep; N1-3, non-rapid eye movement sleep 1–3; SBP, systolic blood pressure; DBP, diastolic blood pressure; NBPF, nocturnal blood pressure fluctuation; ^a^comparison with mild, *p* < 0.017; ^b^comparison with moderate, *p* < 0.017.

### 3.2 Comparison of sleep structure and nocturnal blood pressure in OSA patients before and after CPAP treatment

The results of the first CPAP treatment showed that after using the ventilator, the patients’ TST, MAI, N1%, and N2% decreased compared with those before using the ventilator, while R% and N3% increased. The blood pressure measurements over two nights showed that, except for the insignificant change in SBP, both DBP and NBPF decreased compared with those before treatment (*p* < 0.05) (Table 2).

**Table 2:** Comparison of sleep structure and nocturnal blood pressure in OSA patients before and after CPAP treatment.

Characteristic	Before (*n* = 477)	After (*n* = 477)	*t*	*p*
TST/m	457.20 ± 74.89	436.66 ± 64.41	5.557	<0.001
MAI/h^−1^	36.62 ± 18.48	16.92 ± 8.99	23.866	<0.001
R/%	16.71 ± 5.62	24.38 ± 6.98	−20.624	<0.001
N1/%	11.67 ± 10.57	6.95 ± 4.88	10.230	<0.001
N2/%	57.09 ± 11.76	45.94 ± 11.43	16.338	<0.001
N3/%	14.53 ± 10.75	22.74 ± 10.20	−15.771	<0.001
SBP/mmHg	117.25 ± 16.49	116.11 ± 17.24	1.249	0.212
DBP/mmHg	77.90 ± 12.43	76.45 ± 12.34	2.433	0.015
NBPF/h	28.83 ± 28.66	10.60 ± 13.92	16.198	<0.001

### 3.3 Effect of CPAP treatment on sleep structure and nocturnal blood pressure in patients with different degrees of OSA

**Figure 2:**
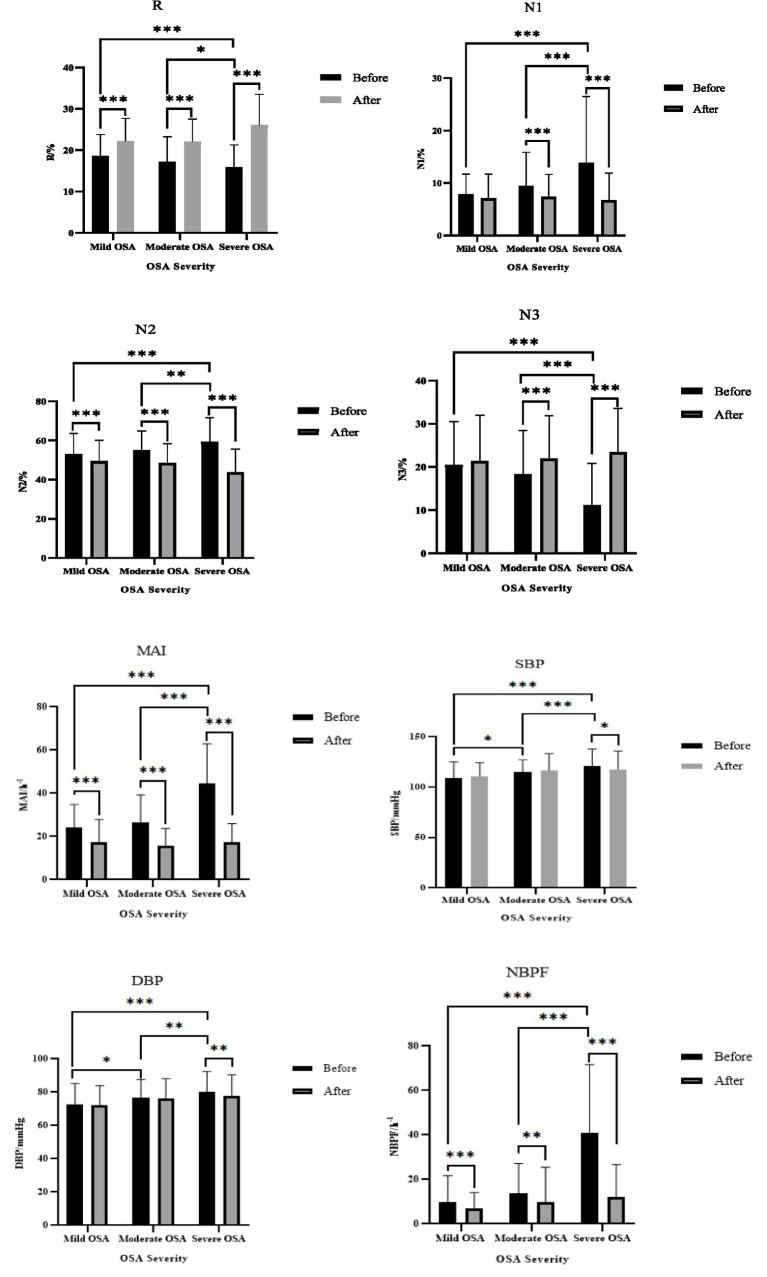
Effects of CPAP treatment on sleep structure and nocturnal blood pressure in patients with different degrees of OSA. ***p < 0.00l; **p < 0.0l; *p < 0.05.

After categorizing OSA patients based on disease severity, we examined the effects of CPAP treatment on sleep structure and nocturnal blood pressure. The results showed that after CPAP treatment, MAI, N2%, and NBPF decreased, while R% increased (*p* < 0.05). For moderate-to-severe OSA patients, N1% decreased and N3% increased after CPAP treatment (*p* < 0.05). Additionally, CPAP treatment significantly improved SBP, DBP, and NBPF in severe OSA patients (*p* < 0.05) (Table 3; Figure 2).

**Table 3:** Effects of CPAP treatment on sleep structure and nocturnal blood pressure in patients with different degrees of OSA.

Characteristic	Mild (*n* = 92)			Moderate (*n* = 108)			Severe (*n* = 277)		
	Before	After	*p*	Before	After	*p*	Before	After	*p*
TST/m	454.90 ± 71.98	439.84 ± 54.66	0.042	452.68 ± 72.10	431.60 ± 66.03	0.009	459.72 ± 77.03	437.59 ± 66.82	<0.001
MAI/h^−1^	24.18 ± 10.69	17.47 ± 10.42	<0.001	26.63 ± 12.74	15.73 ± 7.99	<0.001	44.65 ± 18.24	17.20 ± 8.84	<0.001
R/%	18.63 ± 5.17	22.14 ± 5.56	<0.001	17.22 ± 6.07	22.07 ± 5.45	<0.001	15.87 ± 5.42	26.02 ± 7.48	<0.001
N1/%	7.82 ± 3.90	7.08 ± 4.60	0.113	9.41 ± 6.44	7.37 ± 4.30	<0.001	13.84 ± 12.64	6.74 ± 5.18	<0.001
N2/%	53.05 ± 10.66	49.38 ± 10.65	0.001	55.06 ± 9.79	48.57 ± 9.82	<0.001	59.22 ± 12.34	43.76 ± 11.79	<0.001
N3/%	20.50 ± 10.01	21.40 ± 10.62	0.306	18.31 ± 10.21	21.99 ± 9.90	<0.001	11.07 ± 9.81	23.47 ± 10.14	<0.001
SBP/mmHg	109.41 ± 15.77	110.68 ± 13.73	0.484	114.95 ± 12.27	116.63 ± 16.75	0.321	120.75 ± 17.13	117.71 ± 18.14	0.019
DBP/mmHg	72.74 ± 12.47	72.27 ± 11.58	0.701	76.50 ± 11.18	76.22 ± 11.90	0.814	80.17 ± 12.32	77.92 ± 12.47	0.007
NBPF/h^−1^	9.95 ± 11.78	6.94 ± 7.16	<0.001	13.69 ± 13.51	9.87 ± 15.68	0.009	41.01 ± 30.71	12.10 ± 14.66	<0.001

### 3.4 Correlation analysis between sleep structure and nocturnal blood pressure in OSA patients

Spearman correlation analysis revealed a correlation between sleep quality and nocturnal blood pressure in OSA patients. The R stage was negatively correlated with NBPF, the N1 stage was positively correlated with SBP and NBPF, the N2 stage was positively correlated with NBPF, the N3 stage was negatively correlated with SBP, DBP, and NBPF, and MAI was positively correlated with SBP, DBP, and NBPF (Table 4).

**Table 4:** Correlation analysis between sleep structure and nocturnal blood pressure in OSA patients.

Characteristic	R	N1	N2	N3	MAI	SBP	DBP	NBPF
R	1.0000							
N1	−0.2664^***^	1.0000						
N2	−0.4013^***^	−0.3277^***^	1.0000					
N3	0.1780^***^	−0.4854^***^	−0.5623^***^	1.0000				
MAI	−0.1217^**^	0.2200^***^	0.1181^**^	−0.2816^***^	1.0000			
SBP	−0.0493	0.1241^**^	0.0824	−0.1867^***^	0.1501^**^	1.0000		
DBP	−0.0605	0.0878	0.0773	−0.1394^**^	0.1842^***^	0.7507^***^	1.0000	
NBPF	−0.1092^*^	0.2689***	0.1015^*^	−0.3185^***^	0.5276^***^	0.2527^***^	0.1490^**^	1.0000

^***^p < 0.001; ^**^p < 0.01; ^*^p < 0.05.

Combining the results of correlation analyses, TST, MAI, R, N1, N2, and N3 were used as independent variables, and SBP, DBP, and NBPF were used as dependent variables, respectively, correcting for the effects of age and BMI using multiple linear regression analyses. The results showed that SBP was independently correlated with TST and N3% (*β* = −0.110, *p* = 0.013; *β* = −0.157, *p* = 0.001); DBP was independently correlated with MAI and TST (*β* = 0.145, *p* = 0.002; *β* = −0.088, *p* = 0.048); and NBPF was independently correlated with MAI, N3, and N1 (*β* = 0.375, *p* < 0.001; *β* = −0.143, *p* = 0.001; *β* = 0.092, *p* = 0.026) (Table 5).

**Table 5:** Multiple linear regression analysis of nocturnal blood pressure and sleep structure.

Dependent variable	Independent variable	B	*β*	*t*	*p*
SBP	N3	−0.240	−0.157	−3.482	0.001
	TST	−0.024	−0.110	−2.498	0.013
DBP	MAI	0.098	0.145	3.095	0.002
	TST	−0.015	−0.088	−1.981	0.048
NBPF	MAI	0.581	0.375	9.424	<0.001
	N3	−0.382	−0.143	−3.349	0.001
	N1	0.251	0.092	2.226	0.026

## 4 Discussion

OSA, recognized as a systemic disorder, exhibits cardiovascular complications closely linked to its characteristic acute nocturnal hemodynamic disturbances (17). This study focuses on a core pathophysiological impairment in OSA: nocturnal acute systolic blood pressure surge events (NBPF), defined as the number of events per hour with a systolic blood pressure increase >12 mmHg. These events directly reflect sympathetic nervous system overactivation and vascular endothelial stress, serving as an independent predictor of cardiovascular risk irrespective of mean blood pressure (18, 19). Despite the substantial burden of OSA in China, affecting an estimated 176 million individuals, diagnosis and treatment rates remain critically low (2). CPAP, as the first-line therapy for moderate-to-severe OSA, offers a critical pathway to interrupt this pathological cascade by eliminating respiratory events and suppressing associated microarousals (20).

The current study demonstrated a substantial correlation between the severity of OSA and increased sleep fragmentation (N1%↑, N3%↓) as well as elevated MAI (at baseline, the MAI for severe OSA was 84.66% higher than that for mild OSA). Following CPAP treatment, a significant reduction in the MAI (overall reduction of 53.80%, *p* < 0.05) and an increase in N3% (overall increase of 56.50%, *p* < 0.05) were observed, which served as key indicators of improved sleep architecture. This improvement, especially the increase in N3%, was more significant in patients with moderate to severe OSA (*p* < 0.05). This finding aligns with the CPAP-induced enhancement of slow-wave sleep reported by Chen et al. (21). The key mechanism lies in CPAP’s ability to stabilize the upper airway, thereby interrupting the chain reaction of “respiratory event termination → microarousal” and restoring sleep continuity (22, 23). This finding suggests that MAI may serve as a sensitive electrophysiological marker for assessing CPAP efficacy.

This study employed noninvasive continuous blood pressure monitoring technology to accurately capture NBPF events, circumventing sleep disruption associated with traditional cuff-based measurements. We found that NBPF increased progressively with OSA severity (severe group: increase of 31.27 events/h vs. mild group), corroborating the theory of respiratory event load driving acute cardiovascular stress (24). A single night of CPAP therapy significantly reduced overall NBPF (reduction of 18.23 events/h, *p* < 0.05), with the most pronounced effect observed in severe OSA patients (reduction of 28.91 events/h vs. 2.80 events/h in mild OSA). This finding complements meta-analyses concluding “greater blood pressure improvements with CPAP in severe OSA” (25, 26) and represents the first demonstration that CPAP’s suppressive effect on acute blood pressure events (NBPF) is more pronounced than its modulation of mean blood pressure (SBP/DBP).

A pivotal finding of this study is the elucidation of a quantitative predictive relationship between microarousals (MAI) and acute blood pressure events (NBPF). Multivariate regression analysis demonstrated that MAI serves as an independent predictor of NBPF (*β* = 0.375, *p* < 0.001), indicating that a one-unit reduction in microarousals corresponds to a decrease of 0.375 NBPF events per hour. Furthermore, an increase in N3% exhibited an independent negative correlation with reduced NBPF (*β* = −0.143, *p* = 0.001), suggesting that deep sleep restoration synergistically stabilizes autonomic nervous function. These findings substantiate the pathophysiological hypothesis of “CPAP → MAI suppression → attenuated sympathetic surges → reduction of acute cardiovascular stress (NBPF).” The underlying mechanism involves respiratory event-related microarousals triggering catecholamine surges and abrupt changes in vascular tone, potentially inducing acute systolic blood pressure surges (18, 27).

The empirical data from this study support the adoption of MAI as a core monitoring indicator for the cardiovascular protective effects of CPAP, advocate for NBPF as a priority biomarker for assessing nocturnal cardiovascular risk in OSA patients, and underscore the necessity for prioritized intensive CPAP therapy in severe OSA to prevent acute blood pressure events. Compared to previous studies, the strengths of this work lie in (1) employing noninvasive continuous blood pressure monitoring technology to accurately reflect hemodynamic changes during sleep, (2) utilizing a large-sample severity-stratified design (*n* = 477, severe OSA: 58.1%) to clearly delineate gradient differences in therapeutic efficacy, and (3) being the first to quantify the independent association between MAI and NBPF at the clinical level (β = 0.375), thereby bridging a critical gap in translational mechanism research.

This study has several limitations. First, the assessment was based on a single-night CPAP trial, and the impact of long-term adherence on NBPF was not evaluated. Future studies should incorporate multi-timepoint CPAP follow-up to validate the sustainability of the NBPF suppression effect. Second, pressure titration was not optimized. Building upon this research, further investigation is warranted to determine whether specific CPAP pressure thresholds or critical points exist that maximize improvements in sleep architecture and blood pressure reduction. Saha et al. (14) demonstrated that CPAP at 9 cmH_₂_O (882.6 Pa) improves airflow in stenotic nasal passages and enhances respiratory function by promoting airway patency without exerting excessive pressure on airway tissues. Consequently, it remains to be investigated whether a specific CPAP pressure level or treatment duration could more effectively reduce MAI and NBPF in patients with moderate-to-severe OSA. Thirdly, due to its cross-sectional nature, this study is limited in making causal inferences and cannot establish the temporal sequence of causality between MAI and NBPF.

## 5 Conclusion

In summary, short-term PAP therapy can significantly improve sleep quality in OSA patients, particularly those with severe disease. Its core value lies in the significant reduction of nocturnal systolic blood pressure surges (NBPF) following the suppression of microarousals (MAI). For the first time, this study established MAI as an independent predictor of NBPF (*β* = 0.375) in a large clinical sample. This finding provides an electrophysiological-hemodynamic correlation model for the cardioprotective effects of PAP, offering a quantitative basis for precision intervention in high-risk patients.

## Data Availability

The original contributions presented in the study are included in the article/supplementary material, further inquiries can be directed to the corresponding author.
